# Overexpression of Aquaporin-3 Alleviates Hyperosmolarity-Induced Nucleus Pulposus Cell Apoptosis via Regulating the ERK1/2 Pathway

**DOI:** 10.1155/2022/1639560

**Published:** 2022-04-09

**Authors:** Zetong Zhang, Chen Zhao, Ruijie Zhang, Yiyang Wang, Yanzhu Hu, Qiang Zhou, Pei Li

**Affiliations:** ^1^Department of Orthopedics, Hospital of PLA, Malan 63650, Ürümqi, Xinjiang 841700, China; ^2^Department of Orthopedics, The First Affiliated Hospital (Southwest Hospital) of Army Medical University, Chongqing 400037, China; ^3^Department of Orthopedics, The Third Affiliated Hospital of Chongqing Medical University, Chongqing 400010, China; ^4^Tissue Repairing and Biotechnology Research Center, The Third Affiliated Hospital of Chongqing Medical University, Chongqing 401120, China; ^5^The Second Affiliated Hospital of Chongqing Medical University, Chongqing 400010, China

## Abstract

Intervertebral disc degeneration (IDD) is closely related to osmolarity, which fluctuates with daily activities, and hyperosmolarity may be a contributor to nucleus pulposus (NP) cells apoptosis. Aquaporin-3 (AQP-3) belongs to the family of aquaporins and mainly transports water and other small molecular proteins, which is reduced with the aging of the intervertebral disc. ERK1/2 pathway is one type of mitogen-activated protein kinase (MAPK) and is associated with cellular apoptosis. This study was aimed to investigate the effects of AQP-3 on NP cells apoptosis induced by a hyperosmolarity and focused on the role of the ERK1/2 signaling pathway. We found that NP apoptosis could be induced by hyperosmolarity (550 mOsm/kg), and downregulation of AQP-3 and inhibition of ERK1/2 could be simultaneously observed. Therefore, lentivirus was used to enhance the expression of AQP-3 to compare apoptosis between AQP-3-overexpressed NP cells and the control NP cells. The results showed that apoptosis could be alleviated by overexpression of AQP-3 and the activity of ERK1/2 could also be promoted. Furthermore, we found that the inhibitor U0126 could partly aggravate apoptosis of the AQP-3-overexpressed NP cells. In summary, our results suggested that overexpression of AQP-3 could protect against hyperosmolarity-induced NP cell apoptosis via promoting the activity of the ERK1/2 pathway. This study may shed light on a better understanding of the pathologic mechanism of IDD and bring AQP-3 into the therapeutic approaches for IDD treatment.

## 1. Introduction

Intervertebral disc degeneration (IDD), one of the most important causes of low back pain (LBP), severely affects normal daily life and imposes a significant financial burden on society and the healthcare system [1–3]. Currently, treatments for intervertebral disc degeneration, such as bed rest, functional exercise, physical therapy, and surgery, only relieve pain and do not target the cause of the condition [4–6]. Therefore, an in-depth investigation into the pathophysiological mechanism of intervertebral disc degeneration will provide a theoretical basis for the biological repairment of degenerated intervertebral discs.

Apoptosis, also known as programmed cell death, plays an important role in facilitating IDD by directly reducing the number of nucleus pulposus (NP) cells, thereby affecting the synthesis of extracellular matrix in intervertebral disc NP tissue, such as collagen and proteoglycan [7–11]. The intervertebral disc consists of the upper and lower cartilaginous endplates, the outer annulus fibrosus, and the central NP tissue [12]. NP tissue is highly hydrophilic due to the rich content of negatively charged glycosaminoglycans [13, 14]. Performance of day-to-day activities and changes in the spinal posture cause the NP tissue to be subjected to the constant change in axial compressive stress [15, 16]. This causes water to enter or exit the NP tissue, which in turn causes the in situ osmotic pressure of the NP tissue to fluctuate between 450 mOsm/kg and 550 mOsm/kg, a range that is much higher than the normal osmotic pressure of other extracellular fluids in the human body [17]. Studies by other research groups and our research group have shown that an environment with high osmotic pressure promotes the apoptosis of NP cells [18, 19], but the related mechanism is still unclear.

Aquaporins (AQPs) are transmembrane water channel proteins that can regulate the permeability of cells to water and other small molecules [20, 21]. Previous studies have reported that the expression of AQPs in notochord cells of mouse intervertebral discs is affected by the osmotic pressure environment and is involved in regulating notochord cell differentiation and apoptosis [22]. Aquaporin-3 (AQP-3) is one of the important members of the aquaporin family; it is expressed in rat and human NP tissues and annulus fibrosus tissues. Some studies have proven that AQP-3 expression is significantly reduced in degenerated intervertebral discs compared with normal intervertebral disc [21, 22]. The ERK pathway, one of the important components of the MAPK signaling pathway, participates in the biological processes of various cells. In a study of chondrocytes, ERK1/2 plays a role in osmotic pressure-induced apoptosis, and inhibiting ERK1/2 can increase apoptosis of NP cells in the osmotic pressure culture of intervertebral disc organ models [18, 23]. Studies have also found that AQP-3 can activate the ERK1/2 pathway [19]. The purpose of the current study is to investigate the relationship between AQP-3 expression and apoptosis in NP cells under different osmotic pressure environments and to explore the role of AQP-3 and the ERK1/2 pathway in NP cell apoptosis.

## 2. Methods

### 2.1. Disc Harvest and NP Cell Culture

Twenty-five Sprague-Dawley rats (female, 300–320 g and 12–13 weeks old) were purchased from the Animal Center of Southwest Hospital affiliated with the Army Medical University. The animal care methods were carried out according to the relevant guidelines [SYXK (YU) 2012-0012] and approved by the Ethics Committee at Southwest Hospital affiliated with the Army Medical University. After rats were sacrificed with excess carbon dioxide inhalation, the lumbar discs were separated under sterile conditions. Then, the innermost NP tissue was harvested under a dissecting microscope. NP cell pellet was obtained after sequential enzymatic digestion with 0.25% trypsin for 5–10 minutes and 0.25% Type II collagenase for 10–15 minutes at 37°C. Thereafter, NP cell pellets were resuspended with DMEM/F12 containing 10% fetal bovine serum (FBS) and 1% penicillin-streptomycin and were cultured under standard conditions (37°C, 21% O_2_, and 5% CO_2_). The passage 2 (P2) NP cells were used in this study. The medium osmolarity levels of 330 mOsm/kg and 550 mOsm/kg were used in this study and these defined osmolarity levels were adjusted with sodium chloride and verified with a freezing-point osmometer (FM-8P, Shanghai Medical College Instrument Co. Ltd, China).

### 2.2. Cell Transfection

Briefly, after NP cells seeded in the 6-well plate were grown to approximately 50–55% confluence, NP cells were incubated with the recombinant lentiviral vectors LV5-AQP-3 (GenePharma, Shanghai, China) for 48 hours to enhance AQP-3 expression (NP-AQP-3). The control NP cells were transfected with negative vectors (NP-AQP-3-NC). To further purify the transfected NP cells, all transfected NP cells were incubated with a culture medium containing additional puromycin (1 ug/ml) for 5-6 days. The transfection efficacy was verified by observation under a fluorescence microscope, real-time PCR, and western blot assays.

### 2.3. CCK-8 Assay

NP cell proliferation was measured with a Cell Counting Kit-8 (CCK-8, Beyotime, China). Briefly, 2 × 10^3^ cells/well were seeded in a 96-well plate and incubated in a 5% CO_2_ incubator at 37°C with the osmolarity of 330 mOsm/kg and 550 mOsm/kg, and then 20 ul of CCK-8 was added to each well after 12 h, 24 h, 48 h, and 72 h, respectively. Next, after incubation for another 2 h, the potency of cell proliferation indicated by the absorbance at a wavelength of 450 nm was detected.

### 2.4. Flow Cytometry

After being incubated in the osmolarity of 330 mOsm/kg and 550 mOsm/kg, NP cells were harvested with 0.25% trypsin without EDTA and washed 3 times with phosphate buffer solution (PBS). Then, NP cell apoptosis was evaluated by Annexin V-APC/PI double staining according to the manufacturer's instructions (KeyGENBioTECH, China). NP cells were suspended in binding buffer and 1 × 10^5^ cells were incubated with 5 ul of Annexin V-APC and 10 ul of PI at room temperature in the dark for 20 min. Apoptotic cells were counted by FACS scan flow cytometer (NovaCyte, US) and the cells were stained as Annexin V(+)/PI(−) and Annexin V(+)/PI(+) were regarded as apoptotic cells in this assay.

### 2.5. Quantitative Real-Time PCR

Total RNA was extracted from NP cells with TRIzol reagent (Invitrogen, USA) according to the manufacturer's instructions. After determinating RNA concentration, extracted RNA was synthesized into cDNA with a reverse transcription kit (Roche, Switzerland). Subsequently, quantitative real-time PCR was performed to quantify the mRNA expression levels of Bax, Bcl-2, caspase-3, and AQP-3 with 40 cycles through a reaction system containing cDNA, primers (Table 1), and SYBR Green Mix (Roche, Switzerland). *β*-Actin was used as an internal reference and the relative gene expressions were calculated as 2^−∆∆Ct^.

### 2.6. Western Blot

To detect the protein level of apoptosis-related molecules (Bax, Bcl-2, and cleaved caspase-3) and AQP-3, a western blot was performed according to the following steps. Total protein was isolated from NP cells using RIPA lysis buffer with PMSF and phosphatase inhibitor, and the concentration of the protein sample was measured with a BCA Protein Quantification kit. The same amount of proteins in each group was subject to SDS-PAGE and transferred to PVDF membranes. After being blocked with 5% skimmed milk at room temperature for 1 h, these PVDF membranes were incubated with primary antibodies (Bax: Proteintech, 60267-1; Bcl-2: Proteintech, 12789-1-AP; cleaved-caspase3: CST, 9661T; AQP-3: Abcam, ab125219; GAPDH: Proteintein, 60004-1-Ig; *β*-actin: Proteintein, 60008-1; ERK1/2: Santa Cruz, sc-292838; p-ERK1/2: Santa Cruz, sc-101761) at 4°C overnight with a dilution of 1 : 1000, then washed with TBST solution 3 times, and incubated with corresponding HRP-conjugated secondary antibodies (Beyotime, diluted 1 : 2000) at room temperature for 2 h. Then, protein bands were detected using the enhanced chemiluminescent system. GAPDH and *β*-actin were used as the internal reference.

### 2.7. Immunocytochemistry

To analyze AQP-3 protein expression difference between 330 mOsm/kg culture and 550 mOsm/kgculture, immunocytochemistry staining was performed on cell slides. NP cells were cultured on cell sides for 3 days, then fixed with 4% paraformaldehyde for 30 min at room temperature, and blocked with 5% BSA for 30 min. The cell sides were incubated with AQP-3 primary antibody (1 : 100, Abcam, US) overnight at 4°C. After being washed 3 times, the cell slides were incubated with the HRP-conjugated secondary antibody (1 : 200, ZSGB-BIO, China) for 1 h. Finally, the color development was finished with DAB and the cell slides were viewed under a microscope (Olympus EX51).

### 2.8. Statistical Analysis

All data were expressed as mean ± SD (standard deviation) and SPSS20.0 software was used for statistical analysis. The difference between two groups was performed by Student's *t*-test. The comparison of multiple groups was performed by one-way analysis of variance (ANOVA), followed by a post hoc test was determined by LSD test. Values of *p* < 0.05 were considered statistically significant.

## 3. Results

### 3.1. Apoptosis of NP Cells Increased under Culture with Hyperosmolarity

**Figure 1:**
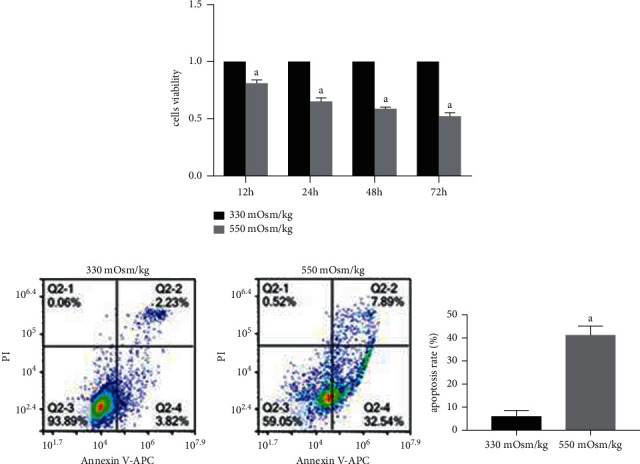
Hyperosmolarity inhibited NP cell proliferation and promoted NP cell apoptosis. (a) The proliferation of rat NP cells detected by CCK-8. (b) Flow cytometry analysis of cell apoptosis rate under different osmotic pressures (330 mOsm/kg and 550 mOsm/kg). Data are expressed as mean ± SD. (A) indicates a significant difference (*p* < 0.05) when compared with 330 mOsm/kg.

CCK-8 assay results showed that compared with NP cells cultured under an osmotic pressure of 330 mOsm/kg for 12 h, 24 h, 48 h, and 72 h, the proliferation of NP cells cultured under an osmotic pressure of 550 mOsm/kg for the same durations was significantly reduced (Figure 1(a)).

**Figure 2:**
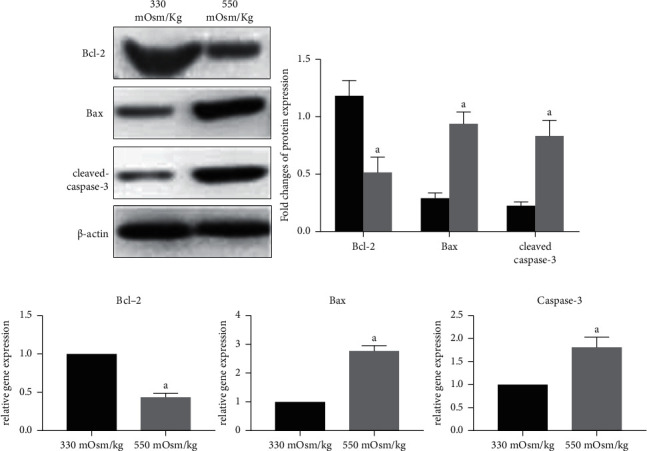
Hyperosmolarity increased the expression of proapoptosis molecules (Bax and caspase-3/cleaved caspase-3) and decreased the expression of antiapoptosis molecules (Bcl-2). (a,b) Real-time PCR and western blotting analysis of proapoptosis (Bax and caspase-3/cleaved caspase-3) and antiapoptosis (Bcl-2) molecules under different osmotic pressures (330 mOsm/kg and 550 mOsm/kg), respectively. Data are expressed as mean ± SD. (A) indicates a significant difference (*p* < 0.05) when compared with 330 mOsm/kg.

Flow cytometry analysis showed that the apoptosis rate was significantly higher in NP cells at osmotic pressure of 550 mOsm/kg than at 330 mOsm/kg (Figure 1(b)). Meanwhile, western blotting and quantitative PCR results showed that compared with an osmotic pressure of 330 mOsm/kg, osmotic pressure of 550 mOsm/kg decreased the mRNA/protein expression of antiapoptotic molecule Bcl-2 and increased the mRNA/protein expression of proapoptotic molecules Bax and cleaved caspase 3/caspase-3 in NP cells (Figures 2(a) and 2(b)). These results suggest that a high osmotic pressure condition promotes apoptosis of NP cells.

### 3.2. AQP-3 Expression in NP Cells Decreased and ERK1/2 Pathway Activity Was Suppressed under a High Osmotic Pressure Condition

**Figure 3:**
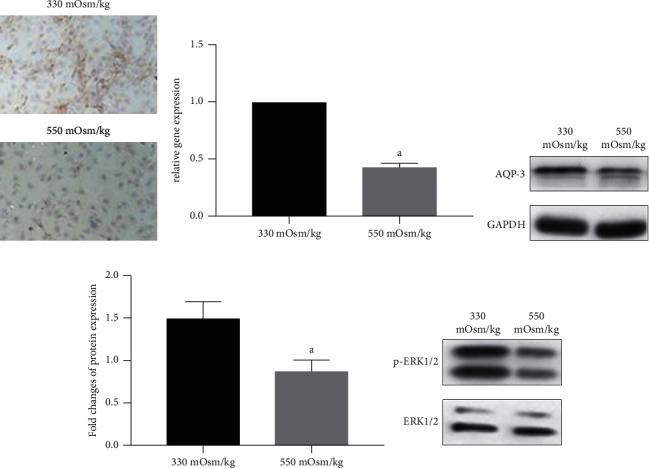
Hyperosmolarity decreased AQP-3 expression and inhibited activation of ERK1/2 signaling in NP cells. (a) Immunocytochemical detection of AQP-3 expression of NP cells under different osmotic pressures (330 mOsm/kg and 550 mOsm/kg). (b, c) Real-time PCR and western blotting analysis of AQP3 expression under different osmolarity (330 mOsm/kg and 550 mOsm/kg), respectively. (d) Western blotting analysis of ERK1/2 activation under different osmotic pressures (330 mOsm/kg and 550 mOsm/kg). Data are expressed as mean ± SD. (A) indicates a significant difference (*p* < 0.05) when compared with 330 mOsm/kg.

Immunocytochemical detection of AQP-3 expression in NP cells under osmotic pressures of 550 mOsm/kg and 330 mOsm/kg showed that, compared to the AQP-3 expression under an osmotic pressure of 330 mOsm/kg, the AQP-3 expression under 550 mOsm/kg was significantly reduced (Figure 3(a)). In addition, western blotting and quantitative PCR results showed that mRNA and protein expression of AQP-3 in NP cells under an osmotic pressure of 550 mOsm/kg were also significantly reduced (Figures 3(b) and 3(c)). These results suggest that a high osmotic pressure environment reduces AQP-3 expression in NP cells. In addition, western blot results showed that, compared to the osmotic pressure of 330 mOsm/kg, the ratio of p-ERK1/2 to ERK1/2 in NP cells decreased at osmotic pressure of 550 mOsm/kg, suggesting that the activity of the ERK1/2 pathway inhibited under a high osmotic pressure condition (Figure 3(d)).

### 3.3. Overexpression of AQP-3 Alleviated Apoptosis of NP Cells in a High Osmotic Pressure Environment

**Figure 4:**
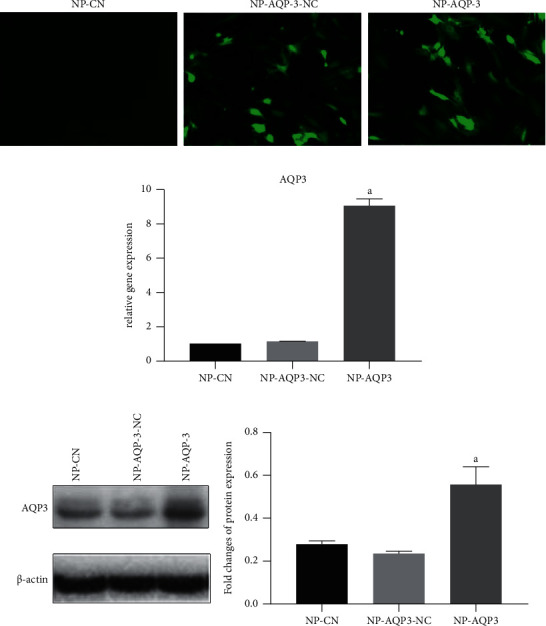
Verification of the efficacy of AQP-3 overexpression. (a) Observation of green fluorescent protein under an inverted fluorescence microscope. (b, c) Verification of the efficacy of AQP3 overexpression in NP cells using real-time PCR and western blotting assays. NP-CN: NP cells without transfection used as the controls. NP-AQP-3-NC: NP cells transfected with negative vectors. NP-AQP-3: NP cells with AQP-3 overexpression. Data are expressed as mean ± SD. (A) indicates a significant difference (*p* < 0.05) when compared with the control group.

Verification of the efficacy of AQP-3 overexpression in NP cells showed that after transfection with a lentivirus overexpressing AQP-3 (NP-AQP-3), the mRNA and protein levels of AQP-3 were significantly higher than those in NP cells transfected with the negative control lentivirus (NP-AQP-3-NC) and NP cells in the blank control group (NP-CN). Furthermore, there was no statistically significant difference in the expression levels of AQP-3 mRNA and protein between NP cells transfected with negative control lentivirus (NP-AQP-3-NC) and NP cells in the blank control group (NP-CN) (Figures 4(a) and 4(b)).

**Figure 5:**
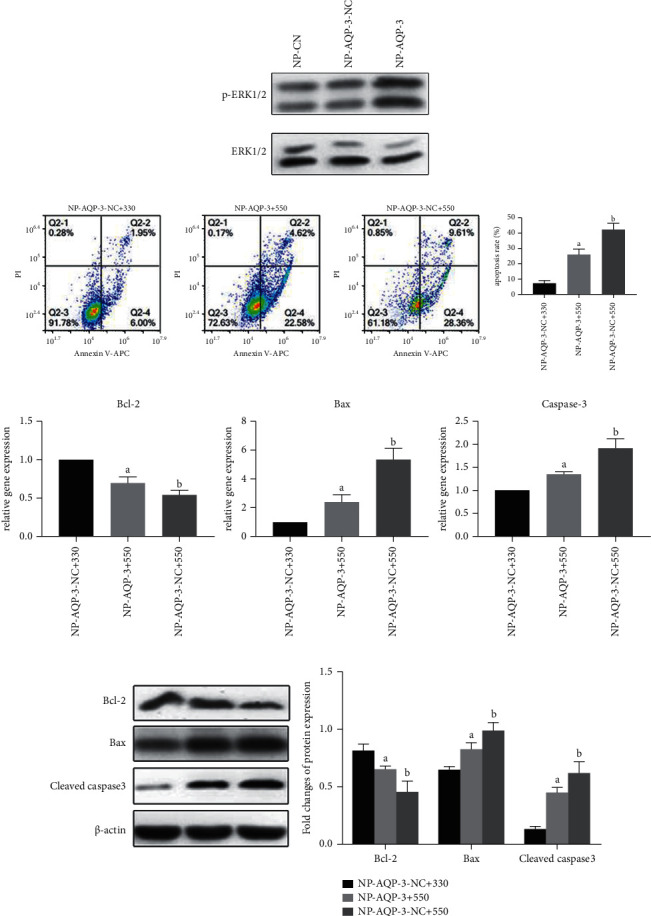
AQP-3 overexpression promoted activation of ERK1/2 signaling and alleviated hyperosmolarity-induced apoptosis. (a) Western blotting analysis of ERK1/2 and p-ERK1/2 expression in AQP-3 overexpressed NP cells under a hyperosmolarity (550 mOsm/kg). (b) Flowcytometry analysis of NP cell apoptosis ratio. (c) and (d): real-time PCR and western blotting analysis of proapoptosis (Bax and caspase 3/cleaved caspase 3) and antiapoptosis (Bcl-2) molecules under different osmotic pressures (330 mOsm/kg and 550 mOsm/kg), respectively. NP-AQP-3 + 330: AQP3 overexpressed NP cells cultured in 330 mOsm/kg. NP-AQP-3 + 550: AQP3 overexpressed NP cells cultured in 550 mOsm/kg. NP-AQP-3-NC + 550: NP cells without transfection (NP-CN) cultured in 550 mOsm/kg. Data are expressed as mean ± SD. (A) indicates a significant difference (*p* < 0.05) when compared with the group of NP-AQP-3 + 330. (B) indicates a significant difference (*p* < 0.05) when compared with the group of NP-AQP-3 + 550.

To observe the change in the activity of the ERK1/2 pathway after AQP-3 overexpression, we further detected protein expression of p-ERK1/2 and ERK1/2 under different osmotic pressure cultures. The western blotting showed that the ratio of p-ERK1/2 to ERK1/2 was promoted in NP-AQP-3 cultured in 550 mOsm/kg compared with NP-CN (Figure 5(a)). Flow cytometry analysis showed that, compared with the apoptosis rate of NP-AQP-3-NC, the apoptosis rate of NP-AQP-3 decreased under an osmotic pressure of 550 mOsm/kg. The western blotting and quantitative PCR results showed that the expression of the antiapoptotic molecule Bcl-2 in NP cells increased, while the expression of proapoptotic molecules Bax and cleaved caspase-3/caspase-3 decreased under an osmotic pressure of 550 mOsm/kg. However, compared with the apoptosis rate under an osmotic pressure of 330 mOsm/kg, the apoptosis rate of NP cells significantly increased, the expression of the antiapoptotic molecule Bcl-2 decreased, and the expression of proapoptotic molecules Bax and cleaved caspase-3/caspase-3 increased under an osmotic pressure condition of 550 mOsm/kg in NP-AQP-3 cells (Figures 5(b)–5(d)).

### 3.4. Apoptosis of AQP-3-Overexpressed NP Cells Increased after Inhibition of the ERK1/2 Pathway

**Figure 6:**
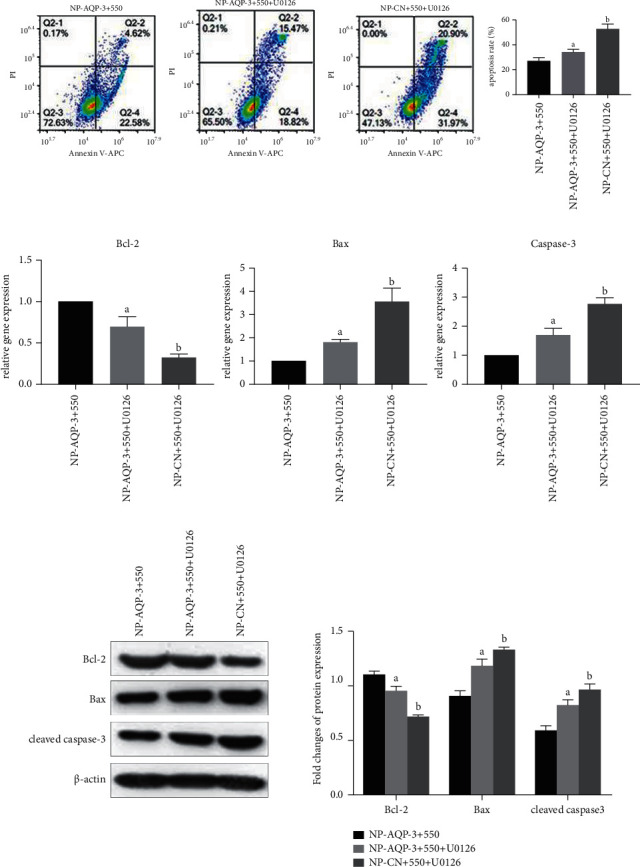
Inhibition of the ERK1/2 pathway promoted apoptosis of AQP-3 overexpressed NP cells under hyper-osmolarity. (a) Flow cytometry analysis of cell apoptosis rate under hyperosmolarity. (b) and (c): real-time PCR and western blotting analysis of proapoptosis (Bax and caspase-3/cleaved caspase-3) and antiapoptosis (Bcl-2) molecules under a hyperosmolarity, respectively. NP-AQP-3 + 550: AQP3 overexpressed NP cells cultured in 550 mOsm/kg; NP-AQP-3 + 550 + U0126: AQP3 overexpressed NP cells cultured in 550 mOsm/kg medium containing U0126; NP-CN + 550 + U0126: NP cells without transfection (NP-CN) cultured in 550 mOsm/kg medium containing U0126. (A) indicates a significant difference (*p* < 0.05) when compared with the group of NP-AQP-3 + 550. (B) indicates a significant difference (*p* < 0.05) when compared with the group of NP-AQP-3 + 550 + U0126.

Under an osmotic pressure of 550 mOsm/kg, the ERK1/2 pathway inhibitor U0126 was added to observe the apoptosis of NP cells after inhibition of the ERK1/2 pathway. Flow cytometric analysis showed that the apoptosis rate was higher in NP-AQP-3 + U0126 cells than that in the NP-AQP-3 group when both of them were cultured under an osmotic pressure of 550 mOsm/kg (Figure 6(a)). The western blotting and quantitative PCR results showed that the expression of the antiapoptotic molecule Bcl-2 in NP cells decreased, while the expression of proapoptotic molecules Bax and cleaved caspase-3/caspase-3 increased after inhibition of the ERK1/2 pathway (Figures 6(b) and 6(c)). Moreover, compared with the NP-CN + U0126 group, the apoptosis rate of NP cells was significantly decreased, the expression of the antiapoptotic molecule Bcl-2 was increased, and the expression of proapoptotic molecules Bax and cleaved caspase-3/caspase-3 was decreased in the NP-AQP-3 + U0126 group under an osmotic pressure of 550 mOsm/kg, indicating that AQP-3 overexpression has certain protective effects (Figures 6(a)–6(c)).

## 4. Discussion

Intervertebral disc degeneration is one of the main causes of LBP. An intervertebral disc consists of three parts, namely, the cartilaginous endplates, the annulus fibrosus, and the NP. Intervertebral disc degeneration occurs due to age, heredity, spinal biomechanics, diabetes, and autoimmunity [24]. The degeneration of the NP tissue mainly manifests by apoptosis and aging of NP cells, reduced synthesis and increased decomposition of the extracellular matrix, and microenvironment changes [25]. Previous studies have reported that high osmotic pressure of 550 mOsm/kg can cause apoptosis-like pathological changes, such as nuclear debris, chromatin condensation, and organelle destruction, while an osmotic pressure environment of 450 mOsm/kg, which is close to the in situ osmotic pressure of the NP tissue, has little influence on the apoptosis of NP cells [18]. Some studies have pointed out that ERK1/2 is involved in the apoptosis of NP cells and that inhibiting the ERK1/2 signaling pathway in these cells will increase the apoptosis rate; it has also been suggested that AQP-3 can activate the ERK1/2 signaling pathway [18].

In this study, we mainly observed the apoptosis of NP cells and the expression of AQP-3 in different osmotic pressure environments and investigated whether AQP-3 participates in the apoptosis of NP cells induced by high osmotic pressure and the role of the ERK1/2 signaling pathway therein. The results of this study showed that a high osmotic pressure significantly promoted the apoptosis of NP cells, reduced the expression of AQP-3, and suppressed ERK1/2 activity. However, AQP-3 overexpression could alleviate apoptosis of NP cells in a high osmotic pressure environment, and apoptosis was increased when the ERK1/2 signaling pathway of cells overexpressing AQP-3 was inhibited. AQP-3, a transmembrane transport protein, carries out water molecule transport by means of an osmotic pressure gradient across the cell membrane [26]. Previous studies have pointed out that AQP-3 plays a role in regulating the apoptosis of certain cells. In recent years, it has been confirmed that AQP-3 could transport H_2_O_2_ in keratinocytes to accelerate the progression of psoriasis [27]. H_2_O_2_ could induce oxidative stress, thereby leading to apoptosis of NP cells, and the overexpression of AQP-3 could alleviate H_2_O_2_-induced apoptosis of rat NP cells [28]. The expression of AQP-3 in NP tissue cells of degenerated human intervertebral discs is significantly lower than that in NP tissue of normal human intervertebral discs [29]. The above research reports suggest that AQP-3 is closely associated with disc NP cell apoptosis.

Our results showed that in the high osmotic pressure environment, the proliferation of NP cells decreased, and the apoptosis increased, as determined by flow cytometry analysis, real-time PCR, and western blot. In previous studies conducted in porcine intervertebral disc organ culture models, our research group also found that the high osmotic pressure environment could reduce the matrix synthesis of NP cells and promote their apoptosis [17]. Similarly, the results of a study by Jiao S et al. are consistent with those of this study, even though 430 mOsm/kg was selected as the osmotic pressure for the control group in their experiments [30]. In this study, we also found that the AQP-3 gene and protein levels in NP cells in a high osmotic pressure environment were significantly reduced. We speculated that this decrease in AQP-3 expression may be related to the apoptosis of NP cells. Previously, Palacio-Manchenoet al. found that after intervertebral discs of C57BL/6 mice were cultured in a hypertonic environment for 14 days, the expression of AQP-3 in notochord cells was upregulated [21], which is inconsistent with the results of this study. Since notochord cells are different from NP cells with respect to sensitivity to osmotic pressure, we speculate that the different cell types used in the studies might lead to different results, but this needs to be verified via further comparative experiments. To investigate whether AQP-3 expression is related to the apoptosis of NP cells under a high osmotic pressure environment, we further generated NP cells overexpressing AQP-3 using a lentivirus with low cytotoxicity and high expression. The results showed that under 550 mOsm/kg, the apoptosis of NP cells was alleviated after AQP-3 overexpression through experiments of flow cytometry, real-time PCR, and western blotting. These results further demonstrated that in a hyperosmolarity environment, the apoptosis of NP cells is closely related to the downregulation of AQP-3 expression, and AQP-3 overexpression in these cells can reduce apoptosis in a hyperosmolarity environment. To further observe the effect of the ERK1/2 pathway, we added U0126 to inhibit ERK1/2 activity. The results of flow cytometry, western blotting, and quantitative PCR suggested that U0126 could inhibit the protective effects of AQP-3 overexpression against a hyperosmolarity-induced apoptosis, suggesting that AQP-3 may regulate apoptosis through the ERK1/2 pathway.

This study also has several limitations. First, this is an in vitro study that investigated the protective effects of AQP-3 on high-osmolarity-induced NP cell apoptosis. If these results are further validated using an in vivo animal model, our study will be improved to a great extent. Second, the rat NP tissue contains NP cells and notochordal cells. Due to the uncertain cellular markers to distinguish NP cells from notochordal cells, researchers cannot assure that there are not any notochordal cells that exist in the NP cells, which are isolated using a routine cell isolation method. In the future, identification of some specific NP cell markers is helpful to obtain pure NP cells, which will avoid some interference caused by the existence of notochordal cells.

## 5. Conclusion

In this study, we observed the apoptosis of NP cells and the AQP-3 expression in different osmotic pressure environments and explored the specific mechanism by which AQP-3 influences a hyperosmotic stress-induced apoptosis in NP cells. Based on the results of this study, we can conclude the following: hyperosmolarity can significantly promote apoptosis of NP cells and reduce the expression of AQP-3; enhancing the expression of AQP-3 in NP cells can alleviate NP cell apoptosis under a hyperosmolarity environment, whereas inhibition of the ERK1/2 pathway partly attenuated the protective effects of AQP-3 against a hyperosmotic stress-induced NP cell apoptosis. This study has revealed the role of AQP-3 in NP cell apoptosis-mediated by a hyperosmolarity environment, which lays a theoretical foundation for further understanding the role of AQP-3 in IDD.

## Figures and Tables

**Table 1:** Primers of target genes.

Gene	Forward (5′-3′)	Reverse (5′-3′)
*β*-Actin	CCGCGAGTACAACCTTCTTG	TGACCCATACCCACCATCAC
Bcl-2	GGGGCTACGAGTGGGATACT	GACGGTAGCGACGAGAGAAG
Bax	GGCGAATTGGCGATGAACTG	CCCAGTTGAAGTTGCCGTCT
Caspase-3	GGAGCTTGGAACGCGAAGA	ACACAAGCCCATTTCAGGGT
AQP-3	AGAAGGAGTTGATGAACCGTTGCG	AACCACAGCCGAACATCACAAGG

## Data Availability

All data were included in this manuscript.
